# Postoperative Airway Management after Submandibular Duct Relocation in 96 Drooling Children and Adolescents

**DOI:** 10.3390/jcm12041473

**Published:** 2023-02-12

**Authors:** Saskia E. Kok, Joris Lemson, Frank J. A. van den Hoogen

**Affiliations:** 1 Department of Otolaryngology-Head and Neck Surgery, Radboud University Medical Center, P.O. Box 9101, 6500 HB Nijmegen, The Netherlands; 2 Department of Paediatric Critical Care, Radboud University Medical Center, P.O. Box 9101, 6500 HB Nijmegen, The Netherlands

**Keywords:** Sialorrhea, cerebral palsy, submandibular duct relocation, postoperative management

## Abstract

The aim of this study was to evaluate our institutions airway management and complications after submandibular duct relocation (SMDR). We analysed a historic cohort of children and adolescents who were examined at the Multidisciplinary Saliva Control Centre between March 2005 and April 2016. Ninety-six patients underwent SMDR for excessive drooling. We studied details of the surgical procedure, postoperative swelling and other complications. Ninety-six patients, 62 males and 34 females, were treated consecutively by SMDR. Mean age at time of surgery was 14 years and 11 months. The ASA physical status was 2 in most patients. The majority of children were diagnosed with cerebral palsy (67.7%). Postoperative swelling of the floor of the mouth or tongue was reported in 31 patients (32.3%). The swelling was mild and transient in 22 patients (22.9%) but profound swelling was seen in nine patients (9.4%). In 4.2% of the patients the airway was compromised. In general, SMDR is a well-tolerated procedure, but we should be aware of swelling of the tongue and floor of the mouth. This may lead to a prolonged period of endotracheal intubation or a need for reintubation which can be challenging. After extensive intra-oral surgery such as SMDR we strongly recommend a extended perioperative intubation and extubation after the airway is checked and secure.

## 1. Introduction

Drooling can be a serious problem in up to 58% of children and adolescents with cerebral palsy (CP) and other neurodevelopmental disabilities [1]. Unintentional loss of saliva may occur as a result of dysfunctional oral control, infrequent swallowing and diminished oral sensitivity [1]. This can cause physical discomfort (i.e., maceration of the skin) as well as emotional consequences (i.e., social isolation and lowered self-esteem). Different treatment options have been suggested in the past, such as oral motor therapy, behavioural treatment, systemic anticholinergic drugs, Botulinum Toxin injections and surgical treatments [1]. Although invasive, surgery is considered to be the most effective therapy to reduce drooling.

Submandibular duct relocation (SMDR) is a commonly performed procedure. During this procedure both submandibular ducts are relocated towards the base of the tongue and most often the sublingual glands are resected simultaneously. It involves relatively extensive floor of mouth surgery. Complications following this surgery may include postoperative pain, swelling of the submandibular gland, floor of the mouth and/or tongue, ranula formation, xerostomia, wound infection, inflammation of a salivary gland (sialadenitis), secondary bleeding or lingual nerve palsy [1,2,3]. Swelling of the floor of the mouth or tongue is a relatively common side effect reported by researchers, which could cause a life threatening airway obstruction in combination with difficult intubation conditions. Reintubation in an emergency setting is already challenging but in combination with severe swelling in the oral cavity and an often spastic, physically and mentally disabled child or young adult might make it impossible. 

Although SMDR could cause a postoperative airway obstruction, most publications only briefly mention this potential life-threatening complication or do not describe it at all. A protocol of the postoperative period is rarely mentioned. To prevent airway obstruction in case of floor of the mouth swelling, children in our centre are admitted to a paediatric intensive care unit (PICU) and stay sedated and intubated for an extended period of time after surgery. Post-operative prolonged intubation protects the airway from obstruction to avoid potentially lethal respiratory problems in these already vulnerable children. Most children that we treat for drooling have physical and/or mental disabilities. They wake up after surgery and often do not understand what is happening. This could result in panic or distress, which may lead to increased movement and agitation. Manual manipulation of the intra-oral wounds or sutures may lead to haemorrhage. Disabilities of these children often make it difficult for them to follow our instructions.

The aim of this study is to investigate the complications of SMDR and evaluate if prolonged endotracheal intubation postoperatively is indicated to secure the airway.

## 2. Materials and Methods

### 2.1. Ethical Considerations

The present study was conducted in accordance with national and international ethical standards, and the Regional Committee on Research Involving Human Subjects approved the study. Before surgery informed consent was provided by caregivers for SMDR.

### 2.2. Patients

This study analysed a historic cohort of children and adolescents who were examined at the Multidisciplinary Saliva Control Centre of the Radboud university medical centre Nijmegen, the Netherlands, between March 2005 and April 2016. Ninety-nine patients had SMDR with simultaneous excision of the sublingual glands for excessive drooling. We excluded 3 patients because of their age (>24 years) [4]. All participants were considered to have a safe pharyngeal phase of swallowing. None of the children had previous surgical procedures of the floor of the mouth or for saliva control. Information was collected concerning age at time of surgery, duration of surgery, postoperative management, duration of intubation, and duration of hospital stay. Occurrence of tongue or floor of the mouth swelling, stridor, postoperative pneumonia and atelectasis were reviewed. Any other complications occurring during surgery or postoperatively were also identified. Co-morbidity was assessed preoperatively by an anaesthesiologist using the ASA physical status. 

### 2.3. Procedure

All surgery were performed under general anaesthesia by the same surgeon. After the papillae of the submandibular ducts were located, the floor of the mouth was infiltrated with Xylocaine 2% with Epinephrine 1:80,000, and an incision was made to create two mucosal islands containing the papilla. The submandibular duct was freed from anterior to posterior, taking special care to prevent damage to the lingual nerve. The sublingual glands were resected bilaterally to prevent ranula formation. After submucosal re-routing of the submandibular ducts to the oropharynx, the papillae were sutured at the base of the tongue with a single stitch, posterior to the glossopharyngeal plica. Meticulous coagulation was performed. We routinely prescribed a 7-day postoperative course of antibiotics (amoxycilline/clavulanic acid) with 5 days of diclofenac for pain management [5]. 

Postoperative management changed in 2006. Before 2006 most patients returned to the ward after surgery. In 2005 a life-threatening complication occurred (airway obstruction due to postoperative haemorrhage), which led to a change in our standard protocol (meticulous bipolar coagulation, local anaesthetics with adrenalin, pre-emptive antibiotics and prolonged intubation with overnight PICU stay). Since 2006 patients were observed overnight at a PICU. Patients remained sedated and (endotracheally) intubated overnight. The next day swelling of the floor of the mouth and tongue was evaluated by a resident ENT by intraoral assessment and in case of absent or minor swelling patients were extubated after weaning. 

### 2.4. Statistical Analysis

Descriptive statistics were employed to summarize patient characteristics. All statistical analyses were performed using SPSS 20.0 for Windows (SPSS Inc, Chicago, IL, USA).

## 3. Results

Characteristics of the 96 patients included for analyses are shown in Table 1. The mean surgical time was 92 min (range 42–193 min), with a mean duration of hospital stay of 4 days (range 2–11 days). All patients were intubated using a nasotracheal cuffed tube. Sixty-two patients received pre-emptive steroids during or after surgery, of the remaining patients 30 did not receive steroids, and of four patients there were no records available. Ninety-three patients received antibiotic treatment during and after surgery, one patient did not receive antibiotics, and of two patients there are no records.

Six of the nine patients who had surgery in 2005, were extubated directly after surgery. Since January 2006 all children (N = 87) were admitted to an PICU with prolonged sedation and intubation after surgery. The mean duration of stay at the PICU was 33 h (range 8–148) with a mean duration of intubation of 24 h (range 3–96). One patient had an unplanned extubation, which was self-inflicted 3 h after surgery.

### 3.1. Complications concerning Airway Obstruction

**Figure 1:**
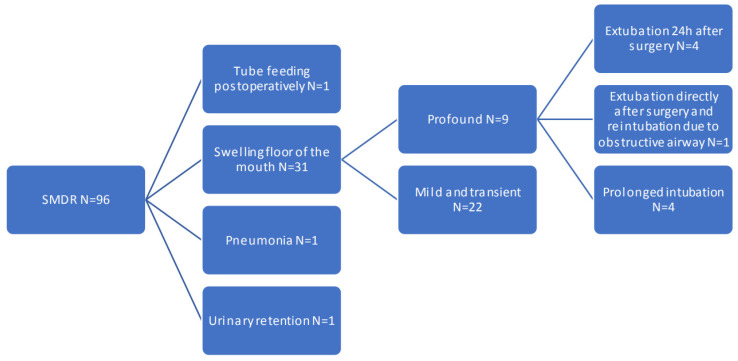
Complications. Tree graph of complications after SMDR.

A tree graph of complications is shown in Figure 1. Swelling of the floor of the mouth or tongue was reported in 31 patients (32.3%). The swelling was mild and transient in 22 patients (22.9%) and profound swelling was seen in nine patients (9.4%). Floor of the mouth swelling was mostly due to oedema. Haematoma was only reported in two patients. Minor bleeding occurred in one patient, which stopped spontaneously without swelling of the floor of the mouth. In one patient there were no records of swelling of the floor of the mouth. 9.7% of the patients who received pre-emptive steroid had profound swelling, 6.7% of the patients who did not receive pre-emptive steroids had profound swelling.

In 2005 one of the patients, who was not admitted to a PICU immediately after surgery, developed profound swelling of the tongue and neck, stridor and increasing respiratory insufficiency within a few hours after surgery. The patient was rushed back to the OR, where an awake flexible nasotracheal intubation was performed and corticosteroids were given. The patient was admitted to the PICU, where he developed a pneumonia (possibly due to aspiration) and stayed on mechanical ventilation for five days. The intraoral swelling subsided and pneumonia was successfully treated with antibiotics. The patient left the hospital 7 days after surgery.

In four out of the nine patients with profound intraoral swelling, prolonged intubation was deemed necessary(4.2% of the 96 patients). These four patients were admitted to the PICU directly postoperatively according to protocol. Three patients had swelling of the floor of the mouth with tongue protrusion and were mechanically ventilated for 48 h, after which the swelling had decreased. During mechanical ventilation one of these patients developed a pneumonia, which was successfully treated with antibiotics. One patient had swelling of the neck and floor of the mouth with tongue protrusion due to a haematoma on the inferior part of the tongue and was mechanically ventilated for four days until the swelling subsided. No reintubations were necessary. 

In four out of the nine patients with profound intraoral swelling, prolonged intubation was not deemed necessary (4.2% of the 96 patients). One patient had sufficient nasal flow/airway after extubation and was observed at the PICU for another 24 h, after which the swelling had settled. In three patients the swelling was more anterior or caudal and did not obstruct the airway.

### 3.2. Other Complications

Overall, patients experienced transient minor eating and drinking difficulties due to pain and discomfort after the surgery. One child had postoperative eating difficulties that necessitated tube feeding for three days. One child developed an atelectasis and postoperative pneumonia. One child experienced urinary retention one week postoperatively, which required suprapubic catheterization. None of the children suffered from ranulas or inflammation of the floor of the mouth. Four patients experienced transient minor nasal bleeding due to the nasotracheal intubation. One patient had a pressure ulcer located at the nose where the nasotracheal tube was positioned.

## 4. Discussion

SMDR is a well-tolerated procedure in children and adolescents. However, in our cohort almost 10% of these patients developed profound swelling of the tongue and floor of the mouth. In half of these cases a compromised airway was observed. Therefore, SMDR seems to justify prolonged postoperative intubation. 

The strength of our study is the study sample size. Our study also has limitations, being the retrospective nature of the study, with a few missing data. We only provide descriptive statistics and did not have a control group. Our main goal of this study was to raise awareness of the risks involved in extensive floor of the mouth surgery related to postoperative airway control.

What is the role of SMDR in the broad spectrum of treatment options for excessive drooling? In our center a multidisciplinary team (a speech and language therapist, a psychologist, a paediatric neurologist, and an otorhinolaryngologist) evaluates all of these patients. In case of sufficient developmental age oral motor therapy and behavioral treatment could provide a good option. In young children who are not suitable for behavioral treatment with Botulinum Toxin injections can be effective. Botulinum Toxin injections reduce the amount of saliva by inhibiting the parasympathetic release of acetylcholine. This has shown to be effective in approximately 50% of children for 6–8 months. Although the effect is temporary, repeated injections are possible. If a more definitive solution is necessary surgery is an option. Our previous research showed that the effect of Botulinum Toxin injections is not a predictor for outcome of SMDR [6]. In our center children with safe oropharyngeal swallowing and non-progressive disease SMDR is the preferred surgical procedure. Due to the risk of aspiration in case of posterior drooling bilateral submandibular gland excision provides a good alternative [7]. If SMDR or submandibular gland excision have insufficient results, parotid duct ligation can be considered in addition.

Previous authors report that submandibular duct relocation with sublingual gland excision is a relatively safe procedure, though complications that could lead to airway obstruction are reported as well. Percentages reported of swelling of the floor of the mouth and airway obstruction vary between 1.4% and 25% [8,9,10,11,12]. Overall transient swelling of the tongue and floor of the mouth is mentioned by many authors. Although most studies describe the minor and major postoperative complications, preventions or treatment are scarcely mentioned. 

Airway obstruction after floor of the mouth surgery can be a serious complication. PICU admittance with prolonged intubation and sedation prevents agitation in these children and can ease caregivers and surgeons’/anaesthesiologists’ worries. Especially reintubation can be extremely difficult. With cerebral palsy being the leading diagnosis in this group of patients they already experience the effects of hypoxia on a daily basis.

On the other hand, prolonged intubation with or without mechanical ventilation has its disadvantages as well. Apart from the high costs associated with an overnight stay at a PICU ward, it also has an impact on parents and caregivers to see their child sedated and intubated on an PICU bed. Prolonged mechanical ventilation is mostly well tolerated, but has risks which could affect the health of the children. Mechanical ventilation could cause atelectasis, pulmonary oedema, pneumonia and (adult) respiratory distress syndrome [13]. With mechanical ventilation there is a need for sedation, which could cause withdrawal or delirium. It is important to avoid unnecessary mechanical ventilation. Also, there are sometimes a limited number of PICU beds available, which could result in OR planning problems or a need to reschedule surgery. 

To date, no studies comparing postoperative PICU and non-PICU admission for SMDR have been performed. Without PICU admittance it is questionable where children after SMDR should be admitted. A child neurology ward has experience with these neurologically disabled children but has little knowledge of airway problems. An Ear, Nose, and Throat ward has knowledge about the procedure and airway problems but has little experience with these vulnerable children often requiring special care. Without a safe alternative, PICU admission might be the only option to maintain a safe airway postoperatively.

A relatively minor bleeding in the floor of the mouth could lead to substantial obstruction of the airway. In case of progressive swelling of the tongue or floor of the mouth a regular transoral intubation quickly becomes impossible. The remaining options in this case are awake flexible nasotracheal intubation or a challenging tracheotomy under local anaesthesia/anaesthetics. Both of these options are not desirable in neurologically disabled, often spastic, children. Cerebral palsy is often caused due to oxygen deprivation at birth. If awake flexible nasal intubation fails, there is a chance that an emergency tracheotomy has to be performed. A second time of oxygen deprivation should be avoided at all cost for both medical as well as emotional reasons. 

The consequences if reintubation and a tracheotomy in a life-threatening situation fail are immense. There is no risk acceptable to possibly lose the life of one of these children. In our series it is reasonable to expect that the 4.2% that could not be extubated after prolonged intubation due to a compromised airway would have been candidates for a challenging reintubation in the event we had extubated them directly postoperatively. To lose a child with such a difficult airway in case reintubation fails is realistic and we can question whether it is justified to take such a risk.

Our study and the literature indicate that there is a reasonable risk of postoperative airway obstruction. In our view, the increased cost and added risk of minor complications associated with PICU admittance outweigh the risk of postoperative oral-oropharyngeal obstruction. As in most day-to-day situations, insurance against low-risk high-damage events is preferable over a higher risk of inconvenience. Therefore, we think postoperative PICU admittance is indicated. 

Implications for Practice

SMDR is an effective treatment to decrease drooling in children and adolescents with developmental disabilities. However, our case series shows a significant risk of postoperative airway obstruction in 9.4% of the patients necessitating prolonged intubation in 4.2%. In order to prevent a life threatening airway compromise we recommend SMDR to be performed by an experienced surgeon with meticulous bipolar coagulation to prevent haemorrhage, local anaesthetics with adrenalin, pre-emptive antibiotics, overnight nasotracheal intubation and sedation. Only extubate the patient after the airway has been carefully checked. In case of profound swelling further prolonged intubation and treatment with steroids should be considered.

## Figures and Tables

**Table 1:** Demographic data.

	No. Patients (Valid %) N = 96
**Gender**	
Male	62 (64.6%)
Female	34 (35.4%)
Age	
Mean (range)	14.9 (6–24)
**Diagnosis**	
Cerebral palsy ᵃ	
Bilateral paresis	61 (63.5%)
Unilateral paresis	4 (4.2%)
Other neurodevelopmental disabilities	31 (32.3%)
**ASA grade**	
1	11 (11.5%)
2	53 (55.2%)
3	30 (31.3%)
Unknown	2 (2.1%)
**GMFCS level (N = 65)**	
I	4 (6.2%)
II	8 (12.3%)
III	19 (29.2%)
IV	21 (32.3%)
V	13 (20.0%)
**Epilepsy**	
Controlled	43 (44.8%)
Intractable	15 (15.6%)
No epilepsy	38 (39.6%)
**Developmental age**	
<4 yrs	50 (52.1%)
4–6 yrs, IQ < 70	18 (18.8%)
4–6 yrs, IQ > 70	3 (3.1%)
>6 yrs	18 (18.8%)
Unknown	7 (7.3%)

ᵃ Confirmed by a paediatric neurologist. GMFCS = Gross Motor Function Classification System, only applicable for children with cerebral palsy; I = Able to walk, II = Difficulty with uneven surfaces, III = Walks with assistive mobility devices, IV = Walking ability severely limited with assistive devices, V = Impaired in all areas of motor function; IQ = Intelligence Quotient.

## Data Availability

The data presented in this study are available on request from the corresponding author. The data are not publicly available due to privacy.
